# The application of a new laminitis scoring method to model the rate and pattern of improvement from equine endocrinopathic laminitis in a clinical setting

**DOI:** 10.1186/s12917-020-02715-7

**Published:** 2021-01-07

**Authors:** A. Meier, J. McGree, R. Klee, J. Preuß, D. Reiche, M. de Laat, M. Sillence

**Affiliations:** 1 Queensland University of Technology (QUT), School of Biology and Environmental Science, Brisbane, Queensland 4000 Australia; 2 Boehringer Ingelheim Vetmedica GmbH, Ingelheim am Rhein, Germany

**Keywords:** Insulin, Laminitis, Equine metabolic syndrome, Obel, Diagnosis

## Abstract

**Background:**

Endocrinopathic, or hyperinsulinaemia-associated laminitis (HAL) is a common and debilitating equine foot disease, and although no pharmacological treatments are registered, several are under development. To evaluate the effect of such treatments, an accurate and consistent method is needed to track the clinical signs of laminitis over time, and the natural history of the disease, in terms of a ‘normal’ pattern of improvement, needs to be understood. This study examined the improvement pattern in clinical cases of naturally-occurring HAL subjected to a range of best-practice interventions, using two different scoring methods. Eighty horses and ponies with suspected HAL were enrolled in a study conducted at 16 veterinary practices across Germany. The severity of laminitis was assessed by independent veterinarians using both the traditional Obel method and a modified Obel method developed by Meier and colleagues. Assessments were made on the day of diagnosis (d 0), then on days 4, 9, 14, 25 and 42 during the intervention period. Pain medications were withheld for 24 h prior to clinical examination in all cases.

**Results:**

Time to marked improvement from laminitis varied between individuals, but was difficult to monitor accurately using the Obel method, with the median grade being 2/4 on days 0 and 4, then 0/4 from d 9 onwards. More subtle changes could be identified using the Meier method, however, and the median scores were seen to follow the form of an exponential decay model in most horses, improving from 8/12 on d 0, to 0/12 on d 25. Within this composite scoring method, considerable variation was observed in the rate of improvement of individual clinical signs, with the average time taken for each sign to reach a median score of 0 ranging from 4 days (foot lift and weight shifting) to 25 days (gait when turned in a circle) across all 80 horses.

**Conclusions:**

The Meier method provides a reliable and consistent method for monitoring the clinical status of horses with HAL, and despite the variability, the pattern of improvement described here should provide a useful benchmark against which individual cases and new treatments can be assessed.

## Background

Laminitis is an equine foot disease that can cause severe lameness and has a high mortality rate (due to euthanasia) [1, 2]. Endocrinopathic laminitis is the most common form of the disease, and several studies during the last decade have strongly associated this with insulin dysregulation (ID) [3, 4], indicating that hyperinsulinaemia is a key pathogenic factor [5, 6]. Accordingly, endocrinopathic laminitis may be more accurately described as hyperinsulinaemia-associated laminitis (HAL) and is the most serious clinical outcome associated with ID in horses.

In 2017, Meier et al. [7] reported a method for inducing HAL in insulin-dysregulated ponies, using a ‘challenge diet’ high in non-structural carbohydrates. Although laminitis was diagnosed in 38% of the animals using the traditional Obel grading method [8], this study revealed some shortcomings in that method, as several animals showed clear signs of discomfort, but did not qualify for the lowest Obel grade of 1. Others have noted that HAL tends to have a slower onset and may present with milder clinical signs that other causes of laminitis [9–11], such as the sepsis-related cases that were used to develop the original Obel method in 1948.

Accordingly, a new laminitis scoring method, known as the ‘modified Obel’ or ‘Meier method’ was developed and validated, showing excellent inter- and intra-observer agreement when used to diagnose the severity of the disease upon first presentation, in both experimental animals and a small number of clinical cases [12]. The new method was based on the long-standing Obel method [8], but was developed to comprise a 3-stage process, which examines five key clinical signs of HAL. These include weight shifting, foot lift, gait at the walk and circle and digital pulse palpation. Each sign receives an individual score, to yield an aggregate score on a scale of 0 to 12. Importantly, these key clinical signs have been shown to differentiate laminitis from other causes of lameness in the horse [13]*.*

The severity of laminitis can be an important a prognostic indicator [14, 15], but despite the value of the Meier method for scoring severity at first presentation, it has not been used previously to monitor the rate of clinical improvement following a bout of laminitis. In fact, as far as we are aware, no one has attempted to describe a typical pattern of clinical improvement from HAL, even though an understanding of the natural history of the disease is critical to the development and assessment of new interventions and treatments.

Thus, the present study aimed to characterize the pattern of improvement from HAL after dietary and other interventions, in a large cohort of horses and ponies over a 42 d period. The assessments were based on grades and scores obtained using both the traditional Obel method and the newer Meier method, to test the suitability of the latter method for monitoring improvement rate in a clinical setting.

## Results

### Laminitis improvement pattern

**Fig. 1:**
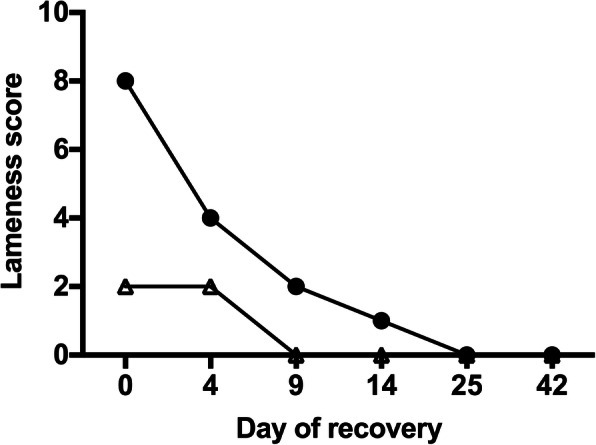
Median laminitis severity scores determined in 80 horses using the Meier (●) and Obel (Δ) methods over a 42-d period post-diagnosis

It was difficult to discern the pattern of laminitis improvement accurately when using the Obel method (Table 1), as the median Obel grade for the 80 subjects remained at 2 from d 0 to d 4, then fell to 0 and remained there from d 9 onwards (Fig. 1). In contrast, the Meier method (Table 2) showed a much clearer pattern, which followed the form of an exponential decay model and did not reach a median score of 0 until d 25 (Fig. 1.).

**Table 1:** The Obel method of laminitis diagnosis and severity grading (Obel, 1948)

Laminitis grade	Grade description
**Normal**	Horse appears sound
**Obel grade 1**	At rest, the horse shifts its weight between the forelimbs; the horse is sound at the walk, but the gait is stilted at the trot in a straight line and on turning
**Obel grade 2**	The gait is stilted at the walk and the horse turns with great difficulty, but one forelimb can be lifted
**Obel grade 3**	The horse is reluctant to walk and one forelimb can only be lifted with great difficulty
**Obel grade 4**	The horse will only move if forced to

**Table 2:** The ‘modified Obel’ or ‘Meier’ method of laminitis diagnosis and severity scoring

Order of examination	Criteria	Description	Points	Given Points
**Stage 1**				
*Examine horse standing*	**Weight shifting**	No weight shifting	**0**	
		Weight shifting – including shifting weight between all feet;	**2**	
		Abnormal time spent lying down; placing forelimbs in front of body		
*Gently lift each foot up and put back down straight away*	**Forelimb lift**	Prompt and willingly maintained (each forelimb)	**0**	
		Reluctant and maintained with difficulty (each forelimb)	**1**	
		Unable to lift foot/resists attempts to lift foot (each forelimb)	**2**	
**Stage 2**				
*Conduct on hard surface Walk horse approx. 30 m side-on to examiner*	**Gait at walk**	Normal gait	**0**	
		Mild - short, stilted gait - still moves willingly	**1**	
		Moderate - short, stilted gait - reluctant/difficult to walk	**2**	
		Severe difficulty walking or unable to walk*	**6**	
		*do not force horse to walk; skip gait at circle and continue with digital pulse		
*Turn on a short lead clockwise and anti-clockwise*	**Gait at circle**	Normal circling	**0**	
		Mild head rise, difficulty when turning, still moves willingly	**1**	
		Moderate, sharp head rise, reluctance/difficulty turning	**2**	
		Severe difficulty turning, slow and clearly painful	**3**	
**Stage 3**				
*All feet must be square on ground*	**Forelimb digital pulse**	Normal - able to palpate, normal magnitude but not bounding	**0**	
		Increased magnitude or bounding digital pulse (each forelimb)	**2**	
			**Total Score**	

**Fig. 2:**
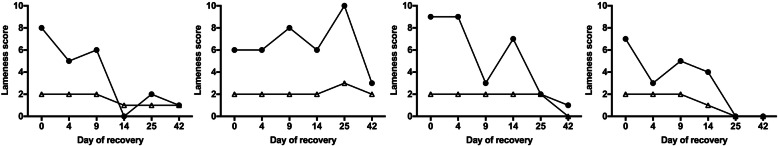
Median laminitis severity scores determined using the Meier (●) and Obel (Δ) methods over a 42-d improvement period, demonstrating an atypical pattern of improvement in four horses (one horse per graph)

A closer examination of this model revealed that although this was a good fit for 80% of the horses (64/80), there were 16 horses (20%) that showed some deviation from this pattern. These were the animals that were slow to improve, or had an uneven rate of improvement, with laminitis scores that increased and decreased over the 42 days. This was more evident when using the Meier method than the Obel method to track improvement, and is illustrated in Fig. 2, which presents four examples of individual horses.

The clearest separation between the group of horses that improved quickly and those that did not, was apparent on d 14. At this time, the median score of 64 animals in the fast improvement group was 1. Of these, 61 animals scored between 0 and 2, and only 3 horses scored a 3. In contrast, the median score of 16 animals in the slow improvement group was 5.5, with a range of 4 to 10. Based on this pattern, the horses were partitioned into ‘fast improvement’ (*n* = 64) and ‘slow improvement’ (*n* = 16) groups for further analysis. The data suggest that the pattern of improvement was not associated with the initial severity of laminitis, as the median (interquartile range; IQR) Meier score for both groups on d 0 was 8 (7–11), with no significant difference between them (*P* = 0.869).

**Fig. 3:**
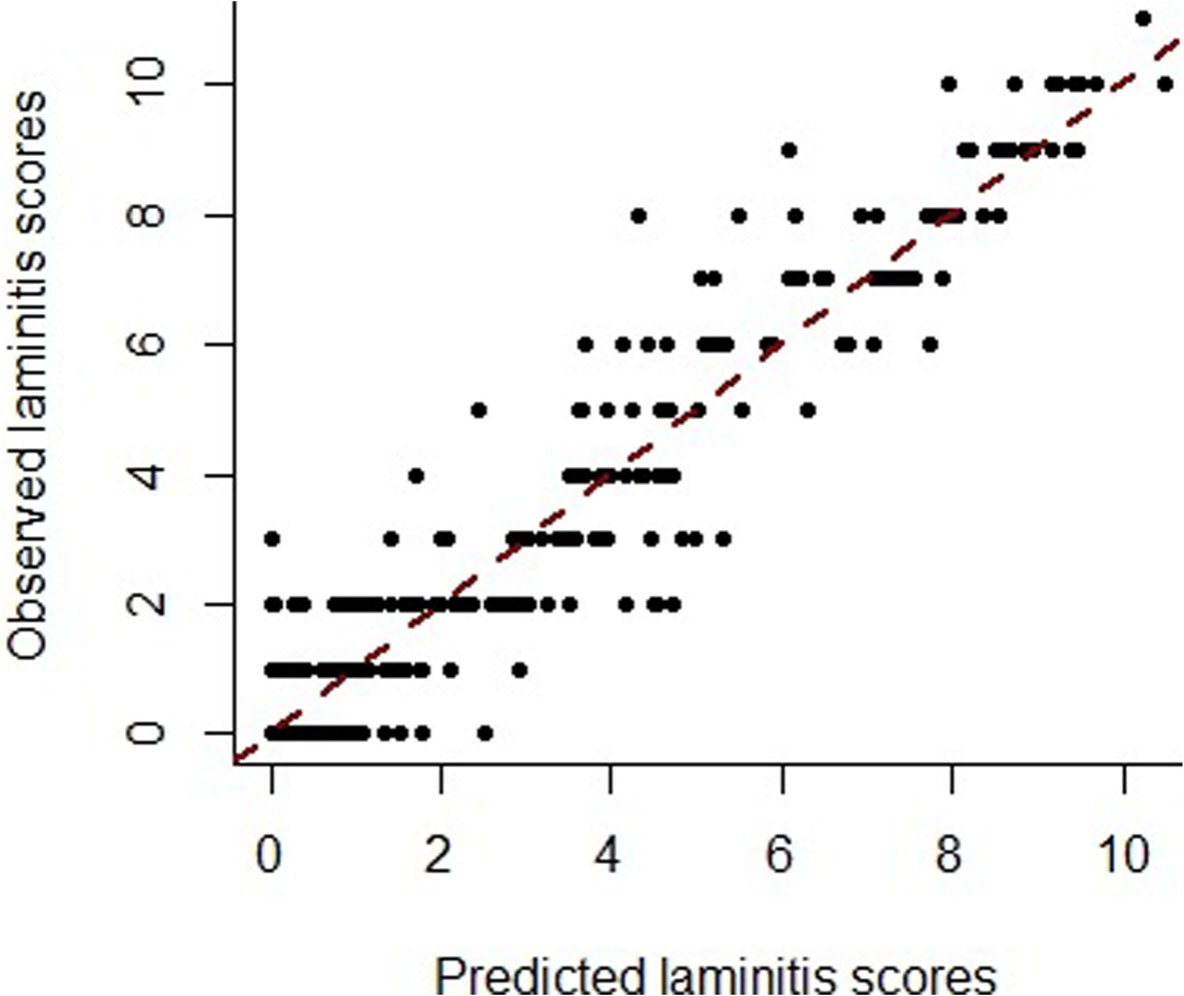
The observed versus predicted scores for the severity of laminitis as measured using the Meier method in 64 horses on 6 occasions during a 42-d improvement period. Predicted values were estimated after fitting an exponential decay model

After removing the animals in the slow improvement group, the exponential decay model was refined using the 64 animals in the fast improvement group, with the best fit being described by the following equation,

$$ {y}_{ij}={\theta}_{0i}\exp \left(-{\theta}_{1i}{t}_{ij}^{\theta_{2i}}\right)+{\epsilon}_{ij}, $$

where *y*_*ij*_ are the Meier scores***,****i* is the *i*th horse and *j* is the *j*th data point***,θ***_***i***_ = (*θ*_0*i*_, *θ*_1*i*_, *θ*_2*i*_)^′^ are a combination of population and individual horse-specific parameters. Specifically, (log *θ*_0*i*_, log *θ*_1*i*_, *θ*_2*i*_)^′^~*MVN*(***θ***, **Ω**) with **Ω** = diag(ω_0_, ω_1_, ω_2_) representing the between horse variability of each parameter. In addition, *t*_*ij*_ denotes the day the *j* th data point was observed on the *i* th horse and *ϵ*_*ij*_~*N*(0, *σ*^2^). The parameter estimates and their variance for the best fitting model are shown in Table 3, with observed and predicted values illustrated in Fig. 3.

**Table 3:** Results from fitting an exponential decay model to Meier laminitis scores measured on days 0, 4, 9, 14, 25 and 42 post-diagnosis in 64 horses and ponies during a clinical study

	Parameter	Estimate	SE	CV(%)
**Fixed effects**	log theta 0	2.07	0.025	1.2
	log theta 1	−1.95	0.158	8.1
	theta 2	1.16	0.081	7.0
	sigma	0.99	0.045	4.6
**Variance of random effects**	Omega 1	0.025	0.007	28
	Omega 2	0.361	0.144	40
	Omega 3	0.087	0.037	42

The estimated value for the average Meier score on d 0 was 7.92, which is close to the observed median Meier score across all horses on this day, and this parameter varied minimally between horses. Lastly, the power term was close to 1 (1.16), suggesting that the decay over time was close to exponential, and this too, showed minimal variation between horses. Overall, this model showed a reasonable fit for all the horses in the fast improvement group, except for some discrepancies when the Meier scores equalled zero, which indicates a potential limitation of the model.

### Improvement in individual clinical signs

The method developed by Meier and colleagues allocates scores to individual clinical signs of HAL, on a scale of 0 to 2 for weight shifting, forelimb lift and forelimb digital pulse; a scale of 0 to 3 for gait at the circle; and 0 to 6 for gait at the walk. The scores for these individual signs were examined using the whole group of 80 horses, to determine the rate of change for each sign, including the entire variation seen in this study (Table 4).

**Table 4:** Median scores for individual clinical signs of laminitis, recorded using the Meier method of severity scoring in 80 horses over a 42-d improvement period

Criterion (max score)	Day of recovery					
	0	4	9	14	25	42
Weight shifting (2)	2	0	0	0	0	0
Forelimb lift (2)	1	0	0	0	0	0
Gait at the walk (6)	2	1	1	0	0	0
Gait at the circle (3)	2	1	1	1	0	0
Forelimb digital pulse (2)	2	2	0	0	0	0

**Fig. 4:**
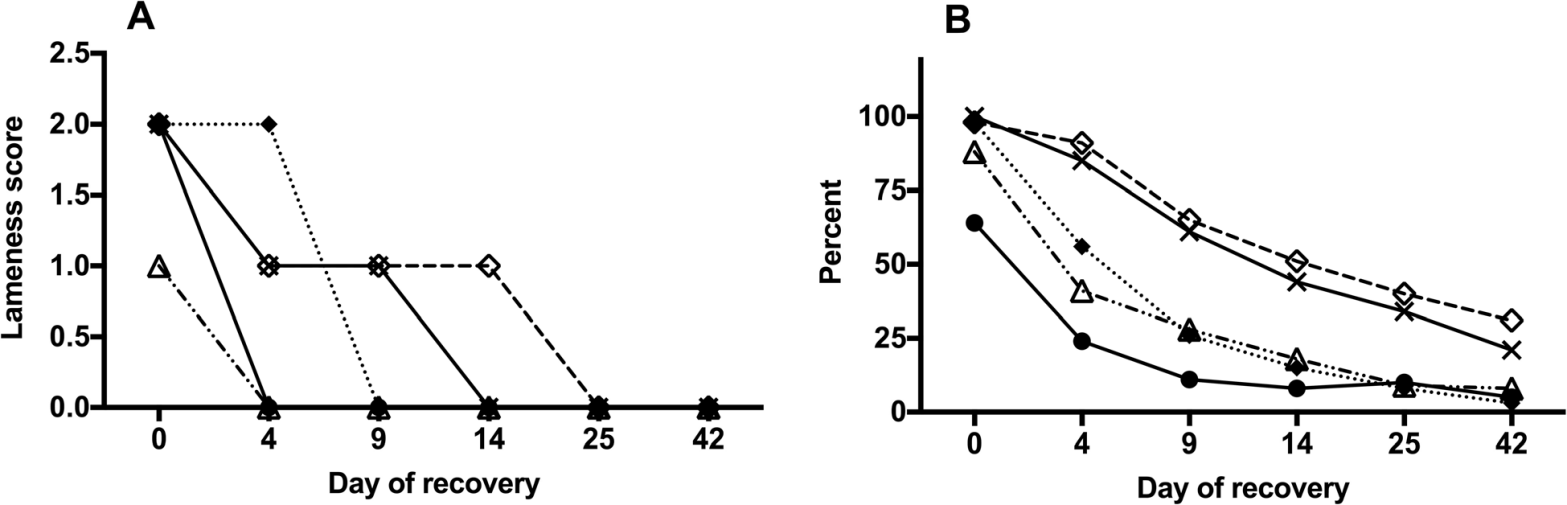
Panel **a**: Median scores for individual clinical signs of laminitis, recorded using the Meier method of severity scoring in 80 horses over a 42-d improvement period. Panel **b**: Percentage of horses with a positive score for each criterion over the same period (Panel **b**). The clinical signs included: weight shifting (●), resistance to lifting the forelimbs (Δ), a bounding digital pulse (♦) atypical gait when walking in a straight line (x), atypical gait when turning in a circle (◊)

As evident in Fig. 4a, the median score for resistance to lifting the feet and weight shifting improved rapidly by d 4 post-diagnosis. In contrast, the median score for bounding digital pulse did not reach 0 until d 9, and the locomotor criteria of gait at the walk and gait while circling only reached a median of 0 on d 14 and d 25, respectively.

The pattern of improvement in the individual clinical signs is further illustrated in Fig. 4b, which shows the percentage of horses with a positive score for each criterion on each occasion. This figure shows that three of the criteria (gait at the walk, gait at the circle, and bounding digital pulse) were present in 98% of horses on the day of laminitis diagnosis. After presenting initially with high frequency, the proportion of horses with a bounding pulse fell to 56% by d 4 and 26% by d 9, showing an overall pattern of decay that provided the closest individual match to the total Meier score (shown in Fig. 1). Whereas weight shifting and resistance to foot lifting were the earliest clinical sign to resolve in most horses, gait at the walk and circle recovered in parallel and were the slowest variables to improve.

## Discussion

To our knowledge, this is the first large study to describe the improvement pattern from the clinical signs of suspected HAL and the results show that the Meier severity scoring method, developed specifically for monitoring the signs of HAL, is suitable to evaluate laminitis improvement, not only in a research setting, but also in clinical studies. The strength of the Meier method is that it allows veterinarians to allocate a severity score over a wide range, between 0 to 12, according to individual clinical criteria that are present in the majority of HAL cases. A broader scoring system improves the ability to observe and monitor a change in scoring and hence clinical improvement and prognosis. In comparison, the strength of the Obel method appears to be in the diagnosis and monitoring of sepsis-related and supporting-limb laminitis, where its utility has been proven in laminitis induction studies [16, 17], with Obel grades showing a good correlation with the severity of histopathological damage to the lamellae [18]. The authors suggest the two scoring methods might be used according to the type of laminitis being studied, because although laminitis shares the common clinical sign of foot pain, it is acknowledged that there are differences in the clinical presentation, diagnosis, treatment, and management, depending on the cause [9, 18].

In the present study, laminitis improvement followed an exponential decay pattern in most of the horses. The initially rapid rate of improvement was due to the early resolution of particular clinical signs, especially digital pulses, weight shifting, and resistance to foot lift. This coincided with a period of dietary modification and forced confinement designed to lower insulin concentrations and minimize further damage to the foot, respectively. Together with the use of supportive pads or bandages, this initial management appears to play a key role in the rate of improvement during the initial phase of acute HAL. There seems to be scope, however, to increase the rate of improvement of the locomotor variables, as it took more than 6 times longer (25 d vs 4 d) for median scores to reach 0 for gait at the circle, compared with weight shifting, or resistance to a foot lift. Furthermore, by d 42 more than 25% of the horses were still not able to circle without showing some degree of lameness. The slow improvement in these signs is expected, as during the examination, locomotion would often elicit the most foot pain. In fact, in a recent study, the loading pattern and the kinetics of the hoof were still different in recovering horses 6 to 12 weeks after laminitis treatment began, compared with healthy controls [19].

Across the full cohort, our data are consistent with a previous survey of horse owners, where the median time to return to soundness was estimated to be 30 days, with an IQR of 14 to 91 days [20]. The large range of improvement rates reported in that survey could have been due to several factors, such as the inclusion of more severe laminitis cases, causes other than HAL, subjective lameness assessments made by the owners, and a greater variation in management. In contrast, in the present study the management during the improvement period was standardised and conformed to best practice. Nevertheless, as the present study demonstrates, improvement from laminitis is not an ‘all or nothing’ event, but a multi-faceted continuum. In this respect, the Meier method has an advantage by incorporating a wider range of possible scores and the ability to identify changes in specific clinical signs over time.

A second area in which there is clearly room for improvement, is in addressing the significant cohort of horses that did not show an exponential improvement, but which demonstrated clinical signs that waxed and waned over the monitoring period. This study demonstrated that variation in improvement pattern was not associated with initial severity of laminitis, but studies to investigate the physical and metabolic variables that may explain why some horses do not improve quickly are ongoing, with the hope that we can inform further intervention strategies. Meanwhile, these cases confirm that improvement from HAL can be slow and variable. Indeed, it should be acknowledged that if significant rotation of the distal phalanx occurs, a horse may never recover fully, and unless the underlying ID is addressed, the horse will continue to be at risk of further laminitis bouts throughout its life [21]. Determining the reasons for slow improvement and/or recurrence is particularly important, as these are associated with a high euthanasia rate [1]. A recent survey indicated that factors such as a previous history of laminitis and the degree of insulin dysregulation are predictive of laminitis recurrence [22], and further detailed investigations are currently underway to determine the association of these and other factors with improvement rates.

Our observation that weight shifting had a lower prevalence (64%) on the day of diagnosis than the locomotive criteria and digital pulse (98–100%), is similar to that seen in an earlier study [2]. This criterion could be confounded by the presence of a different observer, to whom the horse is not accustomed, and/or the removal of a horse from its environment during the examination. As such, it is recommended that stage 1 of the examination should occur prior to removing the horse from its stable or paddock. Nevertheless, there is evidence that stance is a good distinguisher of laminitis-associated lameness [13], and the inclusion of a clinical sign that resolves rapidly may help to differentiate horses that do not recover quickly, allowing early intervention. Similarly, resistance to lifting the forelimbs also provides useful information on the degree of foot pain and was present in a significant proportion of cases (88%) on d 0. This sign has previously been reported in a cross-sectional study to have a prevalence of 52.7% (CI: 48.6–56.8), which is consistent with our results [13].

The fact that 25 veterinarians were used in the present study may be seen as a limitation of the study as it introduced an additional source of variation. Conversely, as this represented the situation in practice, it could be argued that our findings are more representative of real-world circumstances as a result. Furthermore, we have also previously shown that both the Obel and Meier laminitis scoring methods have excellent inter-observer agreement [12]. It is important to note though, that all participating veterinarians received training in both scoring methods prior to the study, and this would not be the situation in normal practice.

Another potential limitation of the present study is the variation in case management beyond d 9, particularly in terms of diet, exercise and ancillary treatments. This was compensated for in part by the large sample size of 80 horses, and further by the fact that all pain medications were withdrawn 24 h prior to each examination. Nevertheless, this was an inherent limitation of this field study, which did not allow management conditions to be replicated exactly for the entire observation period.

## Conclusions

As several lines of inquiry progress towards the development of new pharmacological treatments for ID and HAL [23–25] the need for a widely accepted scoring method and a clear understanding of the natural history of the disease has become more pressing. The results presented here demonstrate that with good management and standard of care, the clinical response does follow a clear pattern in most horses. Some individual variation occurs though, and a proportion of animals do not recover well, or relapse, for reasons that are currently unknown. Overall, this study has provided new information concerning the rate of improvement of specific clinical signs of laminitis and has demonstrated that the Meier method is useful to monitor improvement from HAL in a clinical setting.

## Methods

### Animal selection

Eighty horses and ponies with naturally occurring, suspected HAL were recruited to participate in a randomized controlled field study at 16 veterinary practices in Germany. These were privately owned animals which remained the property of their owners during and after the study. The cohort contained a wide range of breeds, including: Andalusian (1), Appaloosa (1), Crossbreds (6), German Riding Ponies (12), Haflingers (8), Hanovarians (3), Holstein (1) Icelandics (8), Oldenberg (1) Selle Francais (1), Shetland Ponies (18), Welsh Ponies (8), Westphalian (1) and some of indeterminate breed (11). There were 46 females and 34 males, with a mean (±SE) age of 17 ± 0.72 years and a mean BW (estimated using a weight tape) of 395 ± 17.6 kg.

All subjects presented with an onset of laminitis during the past 48 h, with an Obel score of ≥1/4 and a Meier score of ≥5/12 [12]. Horses with identifiable causes of laminitis other than HAL, such as colic or sepsis, were excluded from the study. Evidence of ID, consistent with HAL, included at least one of the following conditions: a high body condition score (≥ 4/5 on the Carroll and Huntingdon scale) [26], fat in specific sites such as the nuchal crest [27], a previous diagnosis of insulin dysregulation according to basal insulin concentrations or dynamic testing, and/or a diagnosis of Pituitary *Pars Intermedia* Dysfunction (PPID). Horses with PPID were only included if they were either untreated (and remained so), or received a constant dose of pergolide for at least 6 weeks prior to the study, and were maintained on the drug throughout the study.

### Study procedures

The horses enrolled in the study were diagnosed with laminitis (designated as d 0) and monitored on days 4, 9, 14, 25 and 42 by 25 experienced equine veterinarians who were trained in both the Obel [8] and Meier [12] scoring methods. Whereas the Obel method assigns a grade of 0 to 4 based on a cluster of clinical signs in each category [8], the Meier method was used to assign a separate score to five clinical signs which included: weight shifting, resistance to lifting the forelimbs, a bounding digital pulse, atypical gait when walking in a straight line, and atypical gait when turning in a circle [12]. These scores were then summed to derive an overall score on a scale of 0 to 12.

The horses were all managed according to best clinical practice. This entailed being confined in a small lot or stable, fed a restricted diet of grass hay only, and fitted with supportive pads or bandages for a minimum of 9 d. Short-acting pain medications, such as flunixin meglumine and phenylbutazone, were administered at the discretion of the attending veterinarian, but were withheld at least 24 h before each laminitis examination.

From d 9 to 42, management was at the discretion of each attending veterinarian, and this included a range of interventions such as altered diet, exercise, and/or corrective hoof care. All interventions were recorded.

### Data analysis

Model fitting was undertaken to analyse the pattern of laminitis improvement over time. This was performed using the SAEMIX package in the R software program [28] where the maximum likelihood estimates of the parameters were obtained via the stochastic approximation expectation maximisation algorithm [29]. Other statistical procedures were conducted using Sigmaplot™ Version 13.0,^1^ with significance set at *P* < 0.05. The data were subjected to a Shapiro-Wilk test for normality, and parametric data are reported as mean ± SE. Non-parametric data are reported as median and range, or IQR, as are the ordinal lameness scores.

Meier scores (*y*_*ij*_) were subjected to a range of mathematical models, with the parameters modelled using a log-scale to ensure that most of the resulting estimates remained positive. When fitting these models, it was assumed that horses were independent and that the Meier scores were continuous and independent within a horse. No prior information was included in the analysis, but various models were compared using the Akaike information criterion (AIC), which can be used for model comparison when the hierarchical structure is the same between the two competing models [30]. An additive plus proportional residual error was also trialled. While this reduced AIC, the resulting residual plots and individual model fits were not deemed satisfactory. Hence, the initial error structure was retained. Model fit was assessed by inspecting plots of the residuals, observed vs predicted values and individual model fits.

Using the whole population of 80 animals, the data were described best by an exponential decay model, but it became apparent that several animals showed a pattern of improvement that deviated significantly from this model. These were horses and ponies that were slow to recover or which showed various clinical signs that waxed and waned over the 42 days. Therefore, the horses were partitioned into two groups according to criteria described in the results section. The exponential decay model was then re-fitted to the 64 horses in the fast improvement group only. The horses in the slow improvement group (*n* = 16) showed no consistent pattern and were not modelled further. An unpaired Mann Whitney U test was used to compare individual variables between these two groups.

To examine the pattern of improvement for individual clinical signs comprised by the Meier method, the animals were consolidated once again into a group of 80. In this way, the full degree of variation observed in this clinical setting was taken into account.

### Fate of the animals

The horses remained with to their owners at the end of the study.

## Data Availability

The full data sets used and analysed during this study are not publicly available due to the fact that individual client owned horses may be identified, but are available from the corresponding author in a redacted form, or with permission of the owners, on reasonable request.

## Abbreviations

AIC: Akaike information criterion
BW: Body weight
CNS: Cresty neck score
d: Day
HAL: Hyperinsulinaemia-associated laminitis
ID: Insulin dysregulation
IQR: Interquartile range
PPID: Pituitary *pars intermedia* dysfunction
QUT: Queensland University of Technology
SE: Standard error
VICH: Veterinary international conference on harmonization (of technical requirements for registration of veterinary medicinal products)

## Footnotes

1 Sysstat Software Inc., San Jose, California, USA
